# PROTOCOL: Learner‐educator co‐creation of student assessment in health professional education courses: A scoping review protocol

**DOI:** 10.1002/cl2.1392

**Published:** 2024-03-20

**Authors:** Laura A. Killam, Rylan Egan, Christina Godfrey, Amanda Ross‐White, Pilar Camargo‐Plazas, Mercedes Lock, Marian Luctkar‐Flude

**Affiliations:** ^1^ School of Nursing Queen's University Kingston Ontario Canada; ^2^ Health Sciences, Nursing and Emergency Services Cambrian College Sudbury Ontario Canada; ^3^ Queen's Collaboration for Health Care Quality: A JBI Centre of Excellence Kingston Ontario Canada; ^4^ Bracken Health Sciences Library Queen's University Kingston Ontario Canada

## Abstract

This is a protocol for a Campbell Review following JBI scoping review methodology. The objectives are to answer the following questions: What has been reported in the literature about collaborative learner‐educator design, implementation, or evaluation of learner assessment in health professional education? (1) Where is learner‐educator co‐creation of assessment occurring? (i.e., which disciplines, course types, level of learner, year of study). (2) What course assessment decisions are influenced or being made together? (i.e., assessment instructions and/or grades). (3) How much influence do learners have on decision‐making? (i.e., where does it fall on Bovill and Bulley's ladder of participation). (4) How do learners and educators go about making decisions together? (i.e., discussion or voting, with a whole class or portion of the class). (5) What are the perceived benefits, disadvantages, barriers, and/or facilitators reported by the authors?

## 1 BACKGROUND

### 1.1 The problem

There is growing interest in inclusive and empowering ways of teaching in health professional education. Teacher‐directed approaches in education where knowledge is transmitted from experts to passive students such as through lecture and memorizing textbooks may create excessive cognitive load and be ineffective for preparing learners for applied tasks in health professional education (Salari, 2018; Spicer, 2018), particularly when they are overused or fail to engage learners. Educational reformers throughout history have advocated for learner‐engaged approaches to education where the goal is to develop learner metacognition through involving learners more actively in knowledge discovery and problem‐solving (Dewey, 1938; Freire, 1973; Giroux, 2020; Thomas, 2022). Several approaches for actively engaging learners in their education have been integrated into health professional education. For example, collaborative problem‐solving may occur during clinical placements, simulation, or other interactive teaching strategies. Another approach is to engage learners as partners in decision‐making.

For health professional learners, shared decision making with patients, interprofessional teams, and other key stakeholders following graduation has been argued to be a critical skill needed for patient‐centered care (Cribb, 2017). Cribb (2017) argued that for medical graduates to be prepared to make joint decisions about how to solve healthcare problems with key stakeholders after graduation it is important to embed opportunities to learn how to co‐create during their education.

The term co‐creation is used in a variety of ways to describe shared decision making with different parties. Co‐creation in the healthcare context has involved patients, healthcare professionals, and other key stakeholders in collaborative problem solving (Cribb, 2017). Working with others who are more knowledgeable than the learner in an interactive way is theorized to enhance learner development (Vygotskiĭ, 1978). For example, nursing and midwifery learners have been found to gain confidence, improve relationships, and develop their skills when they work with service users and carers to make decisions (O'Connor, 2021). This collaboration often occurs in a clinical context. Unfortunately, it is not possible to guarantee that learners will have the opportunity to co‐create during clinical placements. Embedding co‐creation in courses has potential to reach more learners.

Learner‐educator co‐creation may occur during course planning, discussions, activities, and assessment. The intent of sharing decision‐making power with learners is to engage them in reflection, develop critical thinking skills, and improve learner preparation for practice (Dyson, 2018; Killam, 2023). Other benefits of active learner participation in designing courses may include improved relationships, enhanced learner confidence, more depth in learning, improved academic performance, and development of collaboration skills (Bovill, 2011, 2020a, 2020b; Doyle, 2020; Ha, 2017). These benefits are particularly important for graduates of health professional programs who need to be equipped to be both clinically competent and function in teams. However, learner‐educator co‐creation may be pragmatically challenging within a course due to the time‐consuming nature of participative pedagogy and uncertainty about how to involve learners in curriculum decisions (Bovill, 2011). Concerns have also been raised about the scalability and sustainability of leveraging the potential of partnerships with learners (Barradell, 2021). There remains a need to balance the pressures of industrialized education with the benefits of discursive teaching strategies in health professional education in a way that is feasible.

There are also concerns about equitable access to these partnership opportunities. Unfortunately, opportunities for learner‐educator co‐creation during formal education is often limited to small groups of volunteers (Bovill, 2020b). For example, learners may work outside of a course context to co‐create curriculum or work on research projects. When co‐creation opportunities occur outside of coursework it does not reach all learners in a program and often already socially privileged learners are the ones who benefit (Bovill, 2020b; Mercer‐Mapstone, 2020). Typically, these partnership opportunities are offered to learners who are already highly‐engaged in their education and can afford to participate (Mercer‐Mapstone, 2020). Some learners may not be able to access extracurricular activities (e.g., learners who need to work in order to pay for their education and may therefore not have the time to volunteer). Purposeful action is therefore needed to ensure that all learners enrolled in a program have the opportunity to co‐create with educators (Mercer‐Mapstone, 2020). To maximize the preparedness of health professional graduates for real‐world practice, opportunities for co‐creation may be embedded in learner assessment. Therefore, the focus of this review is on learner‐educator co‐creation of assessment in health professional education that occurs during a course.

### 1.2 Definitions

For the purposes of this review,

- *Learners* are students who are enrolled in a course in an academic context.
- *Educators* are people employed by an academic institution to design and facilitate course(s) and are in a position of power over learners' course grades or progression through the program (including on a pass/fail basis). In literature educators are also called teachers, instructors, professors, tutors, or faculty.
- *Co‐creation* is defined as engaging learners as partners during decision‐making in an interactive way (Bovill, 2020a, 2020b; Killam, 2023). While it overlaps with the goals of other forms of active learning, co‐creation emphasizes learner empowerment and involves a deeper interaction between parties during decision‐making (Bovill, 2020a, 2020b; Martens, 2019).
- *Assessment within a course* is defined as a process whereby a learner's knowledge or skills are inferred through analysis of evidence. During assessment learner work is used to evaluate, measure, and document learner progress or skills in a way that helps to determine if a learner will pass a course. Assessment decisions may include, but are not limited to, planning course outcomes or assessment learning outcomes, instructions, due dates, grading criteria, and/or deciding on final grades.
- *Co‐creation of assessment* is the active involvement of learners in decision making about one or more decisions in relation to planning or completing assessments. It involves sharing control over these decisions with students. In higher education literature, there are several overlapping terms used to refer to co‐creation of assessment such as co‐assessment, co‐design, co‐construction, co‐production, consensus grading and more.
- *Health professional disciplines* are defined according to international standards (International Labour Organization, 2012). These professions include medical doctors, nursing, midwifery, complementary medicine, paramedical practitioners, dentistry, pharmacy, physiotherapy, occupational therapy, respiratory therapy, dieticians, nutritionists, audiologists, speech therapists, optometrists, and other allied health programs.
- *Health professional education* includes academic coursework in a program that prepares learners to practice in any health profession. These programs include, but are not limited to, any academic undergraduate or graduate health professions program pre‐ or post‐licensure. It also includes programs that prepare learners to practice in any of the listed professions in countries where licensure is not required.
- *Courses* are units of teaching that last a pre‐determined length of time, are led by educator(s), and have a fixed list of registered learners. This list may be any size. Learners need to complete courses to earn academic credits that count towards earning educational credentials (e.g., a degree).

### 1.3 Learner‐educator co‐creation of assessment

**Figure 1:**
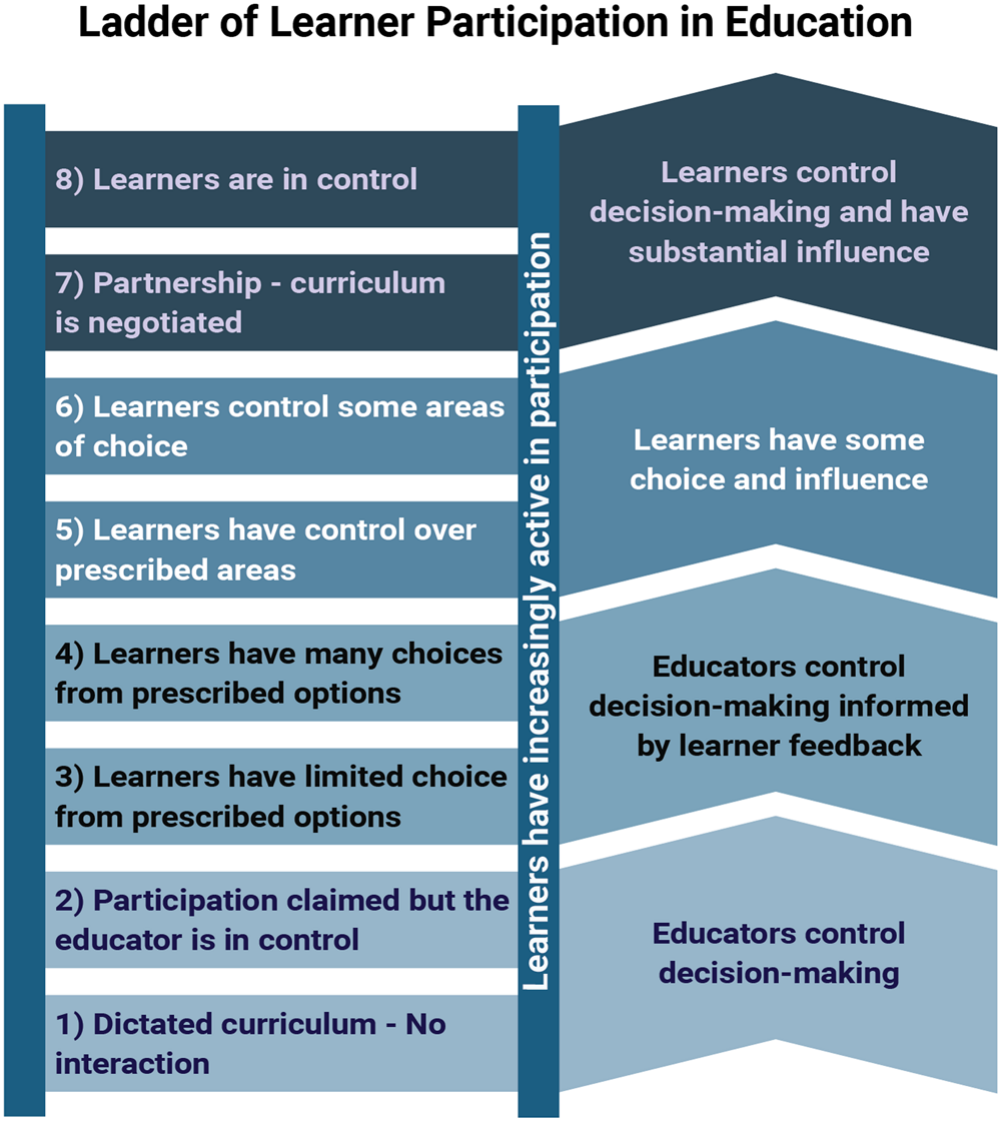
This figure was used with permission from Bovill and Bulley (2011) with minor adaptations from Killam.

Conceptual clarity about what co‐creation involves is complicated by the many terms used to describe it, ambiguity in how it is defined, and misuse of the term in existing literature. Bovill (2011) ladder of participation will be used in this review to differentiate co‐creation from less active forms of learner participation in assessment design (see Figure 1). In this review we will include their top four levels of learner participation where learner influence is limited to specific areas of a course that are pre‐defined by the educator (level 5) or selected by learners (level 6) to where learners partner with educators in negotiating curriculum (level 7) or are in control over decision‐making (level 8) (Bovill, 2011). We are excluding the lower levels of learner participation on this ladder where educators remain in control over planned curriculum (level 1), portray a false sense that learners can influence decisions (level 2), or limit choices to a pre‐defined set of prescribed options (levels 3 and 4). Martens (2019) also conceptualized co‐creation as falling on the upper end of this ladder since learners have more influence on decision‐making during co‐creation than in other forms of participation. During co‐creation, educators do more than listen to student feedback; they focus on empowerment and partnership with learners at a level that is appropriate for the course context and learner readiness (Bovill, 2011; Killam, 2023). The key differentiating feature of studies that we will include is the active involvement of learners in influencing or substantially shaping decisions about learner assessment.

In health professional education co‐creation of assessment has been used in a course context to create simulations with undergraduate nursing and occupational therapy master's learners (Killam, 2021, 2022b; MacKenzie, 2018), determine limited undergraduate nursing exam design characteristics (Killam, 2022a), teach qualitative research at the graduate level (Abboud, 2017), and determine postgraduate nursing learner oral viva grades (Henderson, 2022). Despite the use of co‐creation of assessment in health professional education, ambiguity exists in how educators engage learners in co‐creation within the physical or virtual classroom. A beginning understanding of principles to guide learner‐educator co‐creation have been proposed, but they require refinement based on existing evidence, disciplinary context, and awareness of pragmatic limitations (Killam, 2023). Examining examples of how co‐creation occurs, what decisions are made together, and how those decisions are made is an important step for educators considering co‐creation of assessment in their contexts.

While co‐creation of assessment has potential to reach more learners in a course, it is also complicated by a need to mitigate potential negative consequences of asking students to co‐create (Killam, 2023). For example, the relationship between learners and educators during a course is influenced by the power educators hold over grades. Learners may experience vulnerability during learner‐educator co‐creation of assessment since a productive experience involving honest feedback means exposing their limitations or possibly offending their educator (Johnson, 2020). Learners may therefore want to conceal these thoughts to protect their grades, which are used to assess learner progression through programs and influence scholarships. Also, the way decisions are made may lead to feelings of exclusion and disadvantage already marginalized students if strategies to engage in co‐creation are not well planned (Killam, 2023). Other potentially negative consequences of learner‐educator co‐creation of assessment need to be identified to help educators decide if co‐creation is an appropriate tool for their context.

### 1.4 The need for this review

More evidence about participatory learning approaches is needed to inform institutional policies around learner‐educator co‐creation of assessments in course contexts. Learner‐educator co‐creation in health care higher education contexts may serve to support quality education through enhancing how education meets learner needs, which may support United Nations (2015) Sustainable Development Goal 4 (quality education) (United Nations, 2015). Learner‐educator co‐creation may also be a strategy to help develop learner teamwork, complex problem‐solving, and advocacy skills (Blau, 2017; Dyson, 2018), which are necessary to develop during health professional education to promote a workforce capable of addressing global health challenges, such as advocating for United Nations (2015) Sustainable Development Goal 3 (good health and well‐being).

In this review we have chosen to focus on co‐creation during learner assessment because in higher education literature (1) co‐creation outside of a course has been criticized for failing to be accessible to all learners in a program (Bovill, 2020b; Mercer‐Mapstone, 2020), (2) co‐creation within a course that is not part of assessment may also fail to reach all learners in the course, (3) the stakes for learners are higher during assessment, (4) co‐creation of assessment may be complicated by potential learner vulnerability, and (5) the feasibility of co‐creation related to assessments remains unknown.

A preliminary search of MEDLINE, the Cochrane Database of Systematic Reviews, Epistemonikos, and JBI Evidence Synthesis was conducted (see Supporting Information: Appendix 1). We were unable to identify any published or ongoing scoping reviews about co‐creation in the context of health professional education coursework. A related 2021 systematic review of co‐production (which was identified as an interchangeable term for co‐creation) in nursing and midwifery education was identified during a preliminary search of MEDLINE. The focus of this review of 23 studies published between 2009 and 2019 was on the impact and process of co‐production with a variety of stakeholders in an educational context (O'Connor, 2021). In another review, Barradell (2021) examined literature from 2011 to 2018 using a qualitative synthesis approach to see how the idea of learners as partners was integrated into health professions education. In both of these reviews they included both out of class and in‐class activities. Most of the findings in these reviews focused on co‐creation outside of a course context. Findings specific to co‐creation of assessments were not captured. There are no known current or ongoing systematic reviews or scoping reviews that have described strategies in use for learner‐educator co‐creation of assessment across health professional educational contexts. Owing to the broad nature of our research questions, a scoping review is needed to understand how learner‐educator co‐creation of assessment is implemented, investigated, and reported in healthcare literature.

## 2 OBJECTIVES

What has been reported in the literature about collaborative learner‐educator design, implementation, or evaluation of learner assessment in health professional education?

1. Where is learner‐educator co‐creation of assessment occurring? (i.e., which disciplines, course types, level of learner, year of study)
2. What course assessment decisions are influenced or being made together? (i.e., assessment instructions and/or grades)
3. How much influence do learners have on decision‐making? (i.e., where does it fall on Bovill and Bulley's ladder of participation)
4. How do learners and educators go about making decisions together? (i.e., discussion or voting, with a whole class or portion of the class)
5. What are the perceived benefits, disadvantages, barriers, and/or facilitators reported by the authors?

## 3 METHODS

### 3.1 Inclusion criteria

We will include peer‐reviewed and other academic publications produced globally that outline how learner‐educator co‐creation of assessment is used within health professional education. Inclusion criteria are summarized in Table 1.

**Table 1:** Inclusion and exclusion criteria.

	Inclusion	Exclusion
**Participants (role)**	Learners Educators	Clinical instructors Employers Patients
**Participants (health professional education)**	Enrolled in a program that prepares learners for entry to practice as a healthcare professionals Undergraduate and graduate (i.e., masters or PhD) programs Reports with mixed disciplines (i.e., nursing and arts) in the same course Interprofessional education courses that include health‐care professional learners	Certificate programs Courses for non‐professional healthcare work (i.e., Personal support workers) Professional development courses in employment settings Veterinarian professional education
**Concept (of co‐creation)**	The primary focus of a major section of the report is on co‐creation between learners and educator(s) that involves interaction (i.e., a discussion) The full text of the report includes a description of the co‐creation process or a definition Shared decision may be called co‐creation, co‐construction, co‐design, co‐production, or other terms	No process description or definition (because terms are often misused) Decision making that does not involve both the educator and learners (i.e., collaborative testing) Using learner input (but not involving them in interactive decision‐making) Focused on self‐assessment, feedback, mentorship, or guidance
**Concept (of assessment)**	Any aspect of assessment or evaluation (i.e., determining learning outcomes, assignment instructions, designing assessments, or grading) Learner work that is used to evaluate, measure, and document learner progress or skills in a way that helps to determine if a learner will pass a course (i.e., graded or pass/fail)	Sole focus on: Content curation Designing teaching material Integrating concepts into a curriculum Co‐construction of knowledge
**Context (during a course)**	During an academic course, which includes mandatory and elective courses of any size Learners are enrolled in the course they are making decisions about	Extracurricular activities Curriculum development Volunteer activities Developing material for a future course Research outside of a course Clinical placements Publications with an unclear setting (such as those providing general guidance)
**Evidence types**	All peer‐reviewed research and literature reviews Published commentaries, editorials, theoretical papers, and dissertations Websites of referenced organizations	Study protocols Conference abstracts, proceedings, and posters

#### 3.1.1 Participants

Perspectives of both learners and course educators during health professional education courses will be included in this review. Partners in learner‐educator co‐creation must include both learner(s) and the educator. The report may detail the perspective of one or both participant types. Co‐creation between learners and clients, clinical instructors, people with lived experience, employers, patients, or other experts will be excluded unless the learner and course educator are both also part of the co‐creation process. Participants who are co‐creating during coursework to obtain a certificate, or in hospital or other service provider settings will be excluded. In addition, coursework that is part of obtaining a degree in pure sciences (i.e., biology) will be excluded. We will exclude veterinarian professional education since their practice is with animals.

#### 3.1.2 Concept

Learner‐educator co‐creation of any aspect of student assessment during a course is the concept of interest for this scoping review. To be called co‐creation an effort to share power with learners during the decision‐making process in an interactive way must be described. Learners must also have explicit control or substantial influence over assessment decisions. The kinds of decisions that learners and educators collaborate on may be limited to prescribed areas of the assessment as long as the choices that learners have are not predetermined. Learner‐educator co‐creation during a course may range from influencing one decision about course assessments to making many decisions together. Any situation where an educator invites current learners to substantially influence assessment decisions through collaboration with them that is traditionally only made by an educator will be included.

To be included a major focus of the report must be on learner‐educator co‐creation of assessment that occurs during a course. To be considered a major focus of the report co‐creation must be mentioned in the abstract as part of the aim of the report or main findings. During the search, a broad definition of co‐creation as shared‐decision making in an interactive way will be used. The term co‐creation is often misused (Bovill, 2020b), which means that a definition or description is essential to ensure that co‐creation is interactive and not simply an act of seeking learner input on a decision. Any article that uses the term co‐creation, co‐design, co‐production, co‐construction, consensus grading, or other related terms alongside a definition or description of the process used will be included.

To be included, learner‐educator co‐creation must relate to any aspect of learner assessment during the course. Evaluation is another term that will be included when it refers to making a value judgment about learner work that leads to a grade. There are many kinds of assessment, which may be labeled as objective (sometimes called traditional), performance‐based, and/or authentic assessment. All types of assessment may involve co‐creation and are therefore included in this review.

Publications that say they engaged in collaborative decision making but do not describe or define how decisions were made will be excluded. Publications about co‐construction of knowledge or content curation which do not address course assessment decisions will be excluded. Self‐assessment in the absence of written or verbal dialog with an educator will also be excluded. Articles about providing feedback, mentorship, or guidance will also be excluded unless co‐creation is used in these processes.

#### 3.1.3 Context

The context of the review will be academic course assessment. Learner‐educator co‐creation must occur during the time learners are registered in a mandatory or elective course of any size. The decisions they co‐create must also impact the course that they are registered in.

Studies that describe co‐creation at a curriculum level or where learners volunteer for co‐creation opportunities outside of a course context will be excluded because their involvement impacts future learners (not themselves). Publications about co‐design of educational materials (such as mobile applications or simulations) will be excluded unless it occurs during a course for use in that same course. Co‐creation in a placement or clinical context will be excluded since collaboration is understood as a natural part of these placements and they are not the focus of this review. We have excluded hospital‐based training programs for similar reasons. In addition, learners working as co‐researchers outside of a course context will be excluded. Publications that refer to co‐creation in a variety of contexts will only be used if the use of co‐creation in an academic course context is clearly distinguished from clinical and other contexts. If the source provides general guidance for learner‐educator co‐creation but does not refer to it in the context of a specific course, it will be excluded.

#### 3.1.4 Types of sources

We will be including peer‐reviewed papers, published globally as well as dissertations and editorials, discussion papers or commentaries published in peer‐reviewed journals as well as white papers and websites of known organizations. Excluded are conference abstracts, proceedings, and posters, study protocols, websites, social media posts, white papers and opinions published outside of peer reviewed journals. Study protocols, poster presentations, and conference abstracts will be excluded because they do not provide adequate data to answer the research questions. We excluded other types of evidence due to feasibility. Due to the broad range of terms we are using to identify co‐creation we will need to screen a high number of indexed sources. A previous review about learners as partners in health professional education that was limited to qualitative research noted that they needed to limit their search due to the impractical nature of screening high volumes of literature, which they stated may detract from the meaningfulness of a review's findings (Barradell, 2021). In addition, during preliminary searches of Google and known blog posts the data from these sources was limited in the quality of data that would answer the research questions. Searching for gray literature has also been reported by others to be methodologically problematic since there are issues with search methods, efficiency, and replicability as well as a poor return on the time invested in extracting, assessing, and analyzing data (Adams, 2016).

### 3.2 JBI methodology

The proposed scoping review will be conducted in accordance with the JBI methodology for scoping reviews (Peters, 2020, 2021; Pollock, 2023). JBI methodology was chosen because it is a rigorous and well‐recognized approach for scoping reviews that was developed to address broad questions to investigate the state of knowledge in topics relevant to health professions. The Preferred Reporting Items for Systematic Reviews and Meta‐analyses extension for scoping review (PRISMA‐ScR) checklist will be used to guide the reporting of the review (Tricco, 2018).

#### 3.2.1 Search strategy

The search strategy was designed to locate published information. A three‐step search strategy will be utilized in this review: (1) the initial search to identify keywords, (2), a full search of all databases, and (3) review of relevant reference lists and authors of conference abstracts.

First an initial limited search of JBI, Cochrane, MEDLINE (Ovid), Scopus and Education Source was undertaken to identify articles on the topic. Then, the words contained in the titles and abstracts of relevant articles, and the index terms used to describe the articles were used to develop a full search strategy for Medline (see Table 2) and Education Source (see Supporting Information: Appendix 2). This search strategy, including all identified keywords and index terms, will be adapted for each included database. Then the reference list of all included sources will be screened for additional studies. We will also hand search the authors of conference abstracts or proceedings excluded during full text review. Studies published in any language will be included. No limitation on the date of publication will be used. Gray literature searches will be restricted to dissertations indexed by the respective databases and websites of referenced organizations.

**Table 2:** Search strategy: Medline (via Ovid), December 19, 2023.

Component	#	Query	Results
Participant	1	exp education, dental/or exp education, graduate/or exp education, medical/or exp education, nursing/or exp education, pharmacy/or exp education, public health professional/or exp Students, Health Occupations/	349,196
Participant disciplines	2	exp Health Occupations/	1,870,501
Participant Role	3	(Student* or Learner* or Educator* or Facult* or Preceptor* or Traine*).ti,ab,kf.	664,399
Participant	4	2 and 3	100,918
Participant disciplines	5	(Health profession* or Health science* or Allied health or Medic* or Nurs* or Physiother* or Physical therap* or Occupational Therap* or Podiatr* or Orthoti* or Speech therap* or Speech patholog* or Audiolog* or Prostheti* or Social work* or Paramedic* or Opthalmolog* or Dieteti* or Nutrition* or Psycholog* or Midwif* or Optometr* or Radio* or Pharmac* or dental or “public health”).ti,ab,kf.	6,260,580
Participant	6	3 and 5	318,378
Participant	7	1 or 4 or 6	567,546
Concept (of shared decision making)	8	(co‐construct* or coconstruct* or co‐creat* or cocreat* or consensus or co‐produc* or coproduc* or “democratic educat*” or Enquiry‐based or negotiat* or partner* or “open pedagog*” or “Participative Decision Making” or “student co‐enquir*” or “student coenquir*” or “student partner*” or “student pedagogical team*” or “student voice*” or “Student* as collaborator*” or “Student* as partner” or “student‐faculty collaborat*” or “Student‐faculty partner*” or “student‐staff partner*” or “student‐teacher collaborat*” or “Teacher‐student relationshi*” or Inquiry‐based or “shared creation” or “shared production” or “shared construction”).ti,ab,kf.	466,292
Concept (of assessment)	9	(rubric* or mark* or grade or syllab* or grading or assessm* or evaluat* or formative or summative).ti,ab,kf.	7,173,859
Concept	10	8 and 9	134,676
Participant and Concept	11	7 and 10	7,834
Context	12	(class* or course* or elective or lecture or seminar*).mp.	3,130,069
Participant, Concept, and Context	13	11 and 12	1,789

The databases to be searched include Medline (via OVID, 1946–present), CINAHL (via EBSCOhost, 1981–present), Embase (via Ovid, 1947–present), Education Source (via EBSCOhost, 1929–present), ERIC (via EBSCOhost, 1966–present), and PsycInfo (via OVID, 1806–present). Each database will be searched from the date of inception until present.

#### 3.2.2 Evidence selection

Following the search, all identified citations will be exported and uploaded into Covidence (Veritas Health Innovation) and Endnote 20 (Clarivate Analytics). Duplicates will be removed using Covidence and verified by the lead author. Following a pilot test, titles and abstracts will then be screened by two or more independent reviewers in Covidence for assessment against the inclusion criteria for the review. Full‐text review will also be assessed inside Covidence by two independent reviewers. Reasons for exclusion of sources at full text that do not meet the inclusion criteria will be recorded and reported in the scoping review. Endnote 20 will be used by individual reviewers to make notes on their own articles. Any disagreements that arise between the reviewers at each stage of the selection process will be resolved through discussion, or with an additional reviewer/s. The results of the search and the study inclusion process will be reported in full in the final scoping review and presented in a PRISMA‐ScR flow diagram (Peters, 2021; Tricco, 2018).

#### 3.2.3 Data extraction

Data will be extracted from papers included in the scoping review by two or more independent reviewers using a data extraction tool developed by the reviewers (see Table 3). The lead author will extract data from all included sources. The data extracted will include specific details about the participants, concept, context, study methods and key findings relevant to the review questions. This drafted extraction tool has been piloted by the lead author and will be modified and revised as necessary during the process of extracting data from each included source. Modifications will be detailed in the final scoping review. Any disagreements that arise between the reviewers will be resolved through discussion, or with an additional reviewer. If appropriate, authors of papers will be contacted to request missing or additional data, where required. Although quality appraisal is not a requirement in a scoping review, we will assess the quality of included papers using the appropriate JBI checklist.

**Table 3:** Data extraction instrument.

	Response type
Article number	Numerical
Author	Free text
Year	Numerical
Title	Free text
Country	Free text
Type of information	Selected response: Literature review/Mixed methods/Qualitative/Quantitative/Not research
General aim	Selected response: Process description/Theoretical discussion/Model Development (Evidence based)/Advocacy for co‐creation/Research/Guidelines or advice/Reflection
Specific aim – Describe verbatim	Free text
Study participants	Free text
Research or report design	Free text
Context or setting	Free text
Ethical approval	Selected response: Explicitly obtained/Explicitly not required/Not explicit
Data collection	Free text
Data analysis	Free text
*Article screening checklist – Participants, Concept, Context*	
Student involvement in assessment decisions	Selected response: Yes/No (exclude)
Educator involvement in assessment decisions	Selected response: Yes/No (exclude)
Other persons involvement in assessment decisions – Describe verbatim	Free text
Is decision making power shared in an interactive way	Selected response: Yes/No (exclude)
Are shared decisions about course assessment	Selected response: Yes/No (exclude)
Term(s) used to describe shared decision making – Describe verbatim	Free text
Definition provided – If yes, Describe verbatim	Selected response: Yes/No
Course context	Selected response: Yes/No (exclude)
Health discipline	Selected response: Yes/No (exclude)
*(RQ1) Where is learner‐educator co‐creation of assessment occurring*	
Which disciplines? – Describe verbatim	Free text
Course Type	Selected response: Mandatory/Elective/Unclear/Multiple courses/Likely mandatory
Level of the student	Selected response: Undergraduate/Graduate/Other:
Year of study	Numerical
*(RQ2) What course assessment decisions are influenced or being made together?*	
Evaluation or assessment decisions that are made collaboratively or influenced by students – Describe verbatim (i.e., assessment instructions and/or grades)	Free text
*(RQ3) How much influence do students have on decision‐making?*	
Describe how much influence students have verbatim	Free text
Where does it fall on Bovill and Bulley's (2011) ladder of participation	Selected response: Level 1 through 4 where educators control decision making are excluded from this review/Level 5: Students have control over prescribed areas/Level 6: Students can choose what areas to change/Level 7: There is a partnership and students work with educators to negotiate assessment/Level 8: Students are in control and educators are absent in decision‐making
*(RQ4) How do students and educators go about making decisions together?*	
Describe the overall strategy	Selected response: Discussion/Voting/Consensus/Other:
Can all learners in the class participate?	Selected response: Yes, by choice/Yes, forced/No, small groups are used
Detailed process description	Free text
Considerations to promote feasibility – Describe verbatim	Free text
*(RQ5) Overall Conclusions*	
Benefits – If yes, Describe verbatim	Selected response (Yes/No/Implied) and Free text
Disadvantages – If yes, Describe verbatim	Selected response (Yes/No/Implied) and Free text
Barriers – If yes, Describe verbatim	Selected response (Yes/No/Implied) and Free text
Facilitators – If yes, Describe verbatim	Selected response (Yes/No/Implied) and Free text
*Gaps in the literature*	
Gaps noted by authors – If yes, Describe verbatim	Selected response (Yes/No) and Free text
Limitations noted by authors – If yes, Describe verbatim	Yes/No and Free text

#### 3.2.4 Data analysis and presentation

Data will be displayed in tabular form and accompanied by a narrative summary and/or diagram(s). Table(s) will include details about publication quality, authors, year of publication, country of origin, type (empirical or other), aim(s), participants, design, context or setting, ethics, data collection, data analysis, terminology used, definition(s), where co‐creation is occurring (disciplinary and course context), decision(s) being made, process for decision‐making, and overall author conclusions. This table will be accompanied by a narrative summary and/or diagram(s) may also be used to answer the search questions. We anticipate that descriptive statistics (counts) will be used to identify where co‐creation is occurring (question 1) and what decisions are being made together (question 2). Basic inductive content analysis will be done where appropriate to answer research questions 2 through 5 (Peters, 2021; Pollock, 2023). In basic qualitative analysis categorization is used to aid in the simplification of results to answer review questions. This approach involves immersion in the data, open coding, identifying a coding framework, extracting, organizing, then categorizing data (Pollock, 2023). A diagram illustrating how learners are influencing course assessment decisions will be created if there is sufficient data to do so (question 3 and 4). Further, a summary of the benefits and challenges of co‐creation will be presented graphically or in tabular form where possible (Peters, 2021; Pollock, 2023). Gaps in the literature will also be identified and summarized narratively or in tabular form.

## CONTRIBUTIONS OF AUTHORS

Conceiving the review: LK

Designing the review: LK, RE, CG, ARW, MLF

Developing the search strategy: LK, ARW

Writing the protocol: LK

Providing critical review of the protocol: RE, CG, ARW, PC, ML, MLF

All authors reviewed and approved the final version of the protocol.

## DECLARATIONS OF INTEREST

This review is part of the lead author's PhD in Nursing thesis. As such, she has a vested interest in the timely completion of this review. Some authors have contributed to publications that have been referenced in the background section and may meet the inclusion criteria for the review. These papers will be screened by reviewers who are not named on the paper to reduce bias. None of the authors have a vested interest in the findings of this review.

## SOURCES OF SUPPORT

### Internal sources

- Queen's Collaboration for Health Care Quality: A JBI Centre of Excellence, Canada. Monthly meetings to discuss review methodology.

### External sources

- Social Sciences and Humanities Research Council (SSHRC), Canada.
  The lead author is a recipient of a SSHRC Canada Graduate Scholarship—Doctoral Program.
- Society for Simulation in Healthcare (SSH), USA.
  Funding for a research assistant (ML).
- The Canadian Alliance of Nurse Educators Using Simulation (CAN‐Sim), Canada. Funding for a research assistant (ML) and dissemination.

## Supporting information

Supporting information.

Supporting information.
