# Supplementary material for: PROTOCOL: Learner‐educator co‐creation of student assessment in health professional education courses: A scoping review protocol
Source: Campbell Syst Rev. 2024 Mar 20;20(2):e1392. doi: 10.1002/cl2.1392 (PMC10951880; doi:10.1002/cl2.1392)
Supplement: Supplementary file 1 — Supporting information. [file CL2-20-e1392-s002.pdf]

Appendices

Appendix I: Preliminary Searches

March 24, 2022: Ovid Medline Search Strategy

#	Query	Results
1	(co-creation or cocreation or co-production or co-design or “Open pedagogy” or “Co-construction”).mp.	4,435
2	exp Education, Nursing/	87,351
3	Students, Nursing/	28,108
4	exp Education, Professional/	319,676
5	exp Students, Health Occupations/	80,488
6	exp Students/	151,562
7	or/2-6	420,750
8	1 and 7	128

This search was conducted by ARW. All 128 of these results were imported into Covidence for review by the lead author.

August 10, 2023: Cochrane Database of Systematic Reviews

#	Query	Results
1	(co-creation or cocreation or co-production or co-design or Open pedagogy or Co-construction).mp.	25

Reviewed all titles. One abstract was reviewed. None were in an academic classroom context. There are some about person-centered care and working in partnership with healthcare consumers.

August 10, 2023: Epistemonikos

Query	Results
(title:(co-creation OR cocreation OR co-production OR co-design OR "Open pedagogy" OR "Co-construction") OR abstract:(co-creation OR cocreation OR co-production OR co-design OR "Open pedagogy" OR "Co-construction")) AND (title:(co-creation OR cocreation OR co-production OR co-design OR "Open	59

pedagogy" OR "Co-construction") OR abstract:(co-creation OR cocreation OR co-production OR co-design OR "Open pedagogy" OR "Co-construction")) AND (title:(Student* OR Learner* OR Educator* OR Facult* OR Preceptor* OR Traine*) OR abstract:(Student* OR Learner* OR Educator* OR Facult* OR Preceptor* OR Traine*))	

Reviewed all titles and five abstracts. None of the review articles were in a health professional academic classroom context. O'Connor et al. (2021) is discussed in the introduction of this paper. Some reviews focused on partnerships with other persons in the clinical context or co-production as a strategy to deliver health education.

August 10, 2023: JBI Evidence Synthesis

#	Query	Results
1	(co-creation or cocreation or co-production or co-design or Open pedagogy or Co-construction).mp.	15

Read all titles. Reviewed two abstracts. One had a focus on clinical placements and not on co-creation. The other one was about experience-based co-design but was not in the same context as it focused on acute healthcare services.
