# Supplementary material for: PROTOCOL: Learner‐educator co‐creation of student assessment in health professional education courses: A scoping review protocol
Source: Campbell Syst Rev. 2024 Mar 20;20(2):e1392. doi: 10.1002/cl2.1392 (PMC10951880; doi:10.1002/cl2.1392)
Supplement: Supplementary file 2 — Supporting information. [file CL2-20-e1392-s001.pdf]

Appendix 2. Search strategy: Education Source, January 10, 2024

Component	#	Query	Results
Participant Role	1	AB ((Student* ORLearner* OR Educator*OR Facult* OR Preceptor* OR Traine*)) OR TI ((Student* OR Learner* OREducator* OR Facult* OR Preceptor* OR Traine*)) OR KW ((Student* OR Learner* OR Educator*OR Facult* OR Preceptor*OR Traine*))	1,243,128
Participant disciplines	2	AB ((“Health profession*”OR “Health science*” OR“Allied health” OR Medic*OR Nurse* OR NursingOR Physiother* OR“Physical therap*” OR“Occupational Therap*”OR Podiatr* OR Orthoti*OR “Speech therap*” OR“Speech patholog*” ORAudiolog* OR Prostheti*OR “Social work*” ORParamedic* OROpthalmolog* OR Dieteti*OR Nutrition* ORPsycholog* OR Midwif*OR Optometr* OR Radio*OR Pharmac* OR DentalOR "public health")) ORKW ((“Health profession*”OR “Health science*” OR“Allied health” OR Medic*OR Nurse* OR NursingOR Physiother* OR“Physical therap*” OR “Occupational Therap*”OR Podiatr* OR Orthoti*OR “Speech therap*” OR“Speech patholog*” ORAudiolog* OR Prostheti*OR “Social work*” ORParamedic* OROpthalmolog* OR Dieteti*OR Nutrition* ORPsycholog* OR Midwif*OR Optometr* OR Radio*OR Pharmac* OR DentalOR "public health")) ORTI ((“Health profession*”OR “Health science*” OR“Allied health” OR Medic*OR Nurse* OR NursingOR Physiother* OR“Physical therap*” OR“Occupational Therap*”OR Podiatr* OR Orthoti*OR “Speech therap*” OR“Speech patholog*” ORAudiolog* OR Prostheti*OR “Social work*” ORParamedic* OROpthalmolog* OR Dieteti*OR Nutrition* ORPsycholog* OR Midwif*OR Optometr* OR Radio*OR Pharmac* OR DentalOR "public health"))	434,307
Participant	3	S1 AND S2	117,931

Concept (of shared decision making)	4	AB ((co-construct* OR co-creat* OR consensus ORco-produce* ORdemocratic educat* OREnquiry-based ORnegotiat* OR partner* OR student co enquir ORStudent co inquir ORStudent partner* ORStudent pedagogicalteam* OR student voice*OR Student* ascollaborator* OR Student*as partner OR student-faculty collaborat* ORStudent-faculty partner*OR student-staff partner*OR student-teachercollaborat* OR Teacher-student relationshi* ORInquiry-based ORParticipative DecisionMaking OR "sharedcreation" OR "sharedproduction" OR "sharedconstruction")) OR KW ((co-construct* OR co-creat* OR consensus ORco-produce* ORdemocratic educat* OREnquiry-based ORnegotiat* OR partner* ORstudent co enquir ORStudent co inquir ORStudent partner* ORStudent pedagogicalteam* OR student voice* OR Student* ascollaborator* OR Student*as partner OR student-faculty collaborat* ORStudent-faculty partner*OR student-staff partner*OR student-teachercollaborat* OR Teacher-student relationshi* ORInquiry-based ORParticipative DecisionMaking OR "sharedcreation" OR "shared production" OR "sharedconstruction")) OR TI ((co-construct* OR co-creat* OR consensus ORco-produce* ORdemocratic educat* OREnquiry-based ORnegotiat* OR partner* ORstudent co enquir ORStudent co inquir ORStudent partner* ORStudent pedagogicalteam* OR student voice*OR Student* ascollaborator* OR Student*as partner OR student-faculty collaborat* ORStudent-faculty partner*OR student-staff partner*OR student-teachercollaborat* OR Teacher-student relationshi* ORInquiry-based ORParticipative DecisionMaking OR "sharedcreation" OR "sharedproduction" OR "sharedconstruction"))	158,953
Concept (of assessment)	5	AB ((rubric* OR mark*OR grade OR syllab* ORgrading OR assessm* ORevaluat* OR formative*OR summative*)) OR KW((rubric* OR mark* ORgrade OR syllab* ORgrading OR	765,626

		assessm* ORevaluat* OR formative*OR summative*)) OR TI ((rubric* OR mark* ORgrade OR syllab* ORgrading OR assessm* ORevaluat* OR formative*OR summative*))	
Concept	6	S4 AND S5	28,359
Participant and Concept	7	S3 AND S6	1,963
Context	8	class* OR course* OR elective OR lecture OR seminar*	811,749
Participant, Concept, and Context	9	S7 AND S8	737
